# Supplementary material for: Tumor-Localized Administration of α-GalCer to Recruit Invariant Natural Killer T Cells and Enhance Their Antitumor Activity against Solid Tumors
Source: Int J Mol Sci. 2022 Jul 7;23(14):7547. doi: 10.3390/ijms23147547 (PMC9317565; doi:10.3390/ijms23147547)
Supplement: Supplementary file 1 [file ijms-23-07547-s001.zip › ijms-1777637-supplementary.pdf]

International Journal of *Molecular Sciences*

MDPI

Article

Tumor-Localized Administration of α-GalCer to Recruit Invariant Natural Killer T Cells and Enhance Their Antitumor Activity against Solid Tumors

Yan-Ruide Li, Yang Zhou, Matthew Wilson, Adam Kramer, Ryan Hon, Yichen Zhu, Ying Fang and Lili Yang

[1] Department of Microbiology, Immunology & Molecular Genetics, University of California, Los Angeles, CA 90095, USA; charlie.li@ucla.edu (Y.-R.L.); zzydcat@g.ucla.edu (Y.Z.); mwilson193@g.ucla.edu (M.W.); akramer931@g.ucla.edu (A.K.); ryanhonchenghao@gmail.com (R.H.); yichenzhujohn@g.ucla.edu (Y.Z.); yingfang1913@g.ucla.edu (Y.F.)

[2] Eli and Edythe Broad Center of Regenerative Medicine and Stem Cell Research, University of California, Los Angeles, CA 90095, USA

[3] Jonsson Comprehensive Cancer Center, David Geffen School of Medicine, University of California, Los Angeles, CA 90095, USA

[4] Molecular Biology Institute, University of California, Los Angeles, CA 90095, USA

* Correspondence: liliyang@ucla.edu; Tel.: +13-10-825-8609

Citation: Li, Y.-R.; Zhou, Y.; Wilson, M.; Kramer, A.; Hon, R.; Zhu, Y.; Fang, Y. Tumor-Localized Administration of α-GalCer to Recruit Invariant Natural Killer T Cells and Enhance Their Antitumor Activity against Solid Tumors. *Int. J. Mol. Sci.* **2022**, *23*, 7547. https://doi.org/10.3390/ijms23147547

Academic Editor: Walter Fiedler

Received: 2 June 2022
Accepted: 6 July 2022
Published: 7 July 2022

Publisher's Note: MDPI stays neutral with regard to jurisdictional claims in published maps and institutional affiliations.

Copyright: © 2022 by the authors. Licensee MDPI, Basel, Switzerland. This article is an open access article distributed under the terms and conditions of the Creative Commons Attribution (CC BY) license (http://creativecommons.org/licenses/by/4.0/).

Supplementary Materials

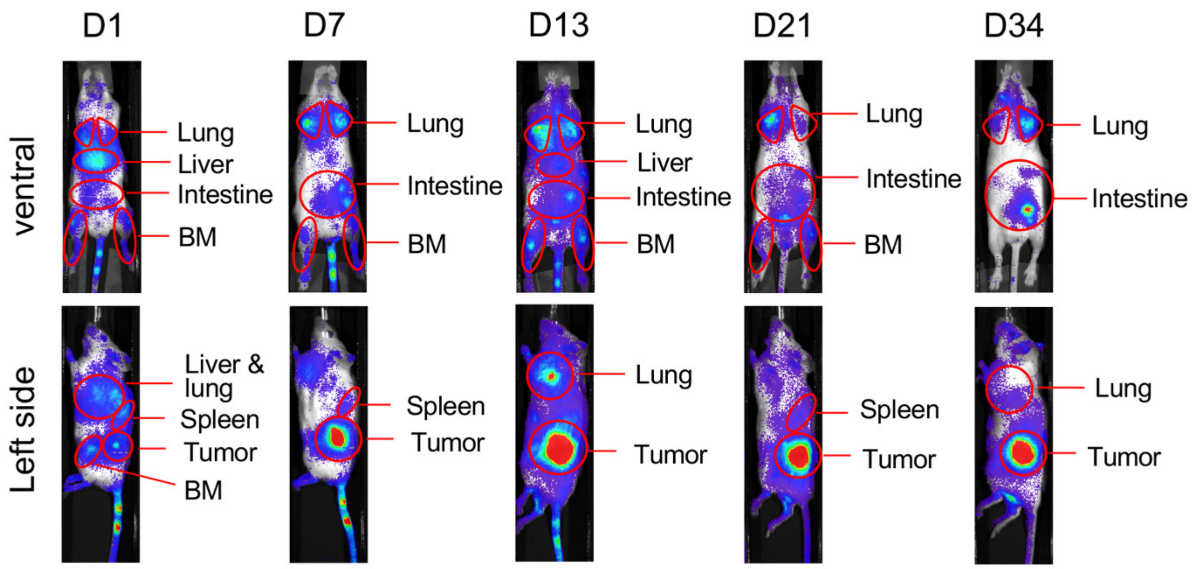

Figure S1. Tissue biodistribution of PBMC-iNKT cells in an A375-CD1d human melanoma xenograft NSG mouse model. Related to Figure 5. Representative images showing iNKT cell load in experimental mice over time. These mice are injected with A375-CD1d tumor cells, αGC and PBMC-iNKT-FG cells. Both ventral and left side views are shown.

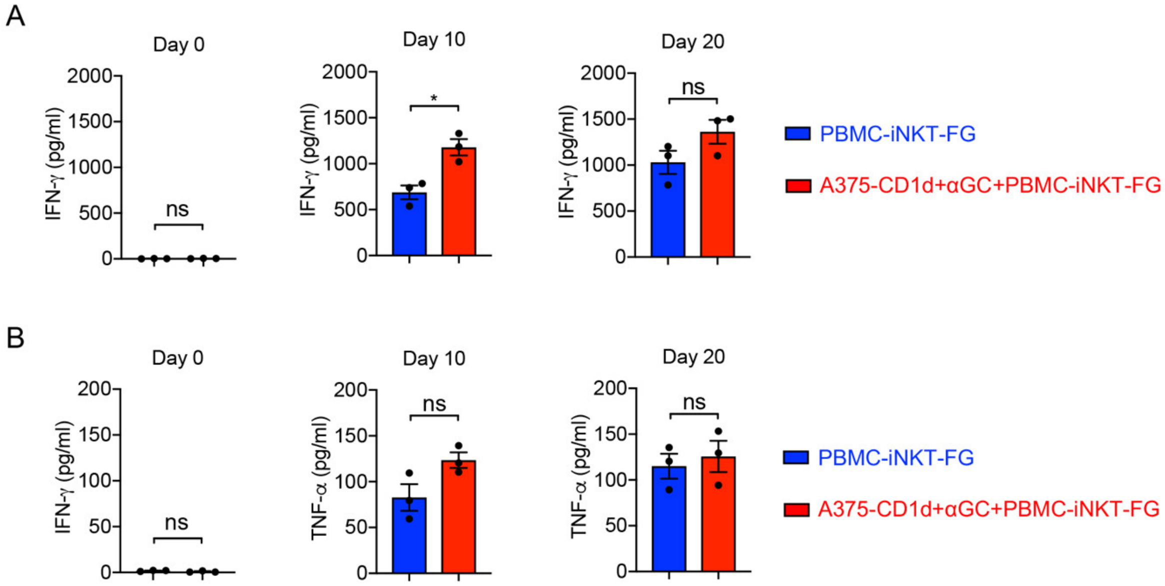

Figure S2. Mouse serum cytokine changes in an A375-CD1d human melanoma xenograft NSG mouse model. Related to Figure 5. Levels of human IFN-γ (A) and TNF-α (B) were measured by ELISA on serum collected from mouse peripheral blood on different days after PBMC-iNKT-FG cell infusion. Representative of 2 experiments. Data are presented as the mean ± SEM. ns, not significant, *$p < 0.05$ by Student's t test.
